# Is COVID-19 Being Used to Spread Malware

**DOI:** 10.1007/s42979-023-01838-6

**Published:** 2023-05-17

**Authors:** Ruqayah N. Ahmed, Amir Javed, Wafi Bedewi

**Affiliations:** 1 Computer Center, University of Samarra, Samarra, Salah Aldin 34010 Iraq; 2 School of Computer Science and Informatics, Cardiff University, Cathays, Cardiff, CF24 4AG UK; 3 Faculty of Computing and Information Technology, King Abdulaziz University, Jeddah, 21589 Saudi Arabia

**Keywords:** Cyber security, Malware, Machine learning, AI, Malicious, COVID-19, Twitter, Tweet, Pandemic

## Abstract

With the rising number of people using social networks after the pandemic of COVID-19, cybercriminals took the advantage of (i) the increased base of possible victims and (ii) the use of a trending topic as the pandemic COVID-19 to lure victims and attract their attention and put malicious content to infect the most possible number of people. Twitter platform forces an auto-shortening to any included URL within a 140-character message called “tweet” and this makes it easier for the attackers to include malicious URLs within Tweets. Here comes the need to adopt new approaches to resolve the problem or at least identify it to better understand it to find a suitable solution. One of the proven effective approaches is the adaption of machine learning (ML) concepts and applying different algorithms to detect, identify, and even block the propagation of malware. Hence, this study’s main objectives were to collect tweets from Twitter that are related to the topic of COVID-19 and extract features from these tweets and import them as independent variables for the machine learning models to be developed later, so they would identify imported tweets as to be malicious or not.

## Introduction

The outrage of the noble coronavirus (COVID-19) in late 2019 has affected all life aspects globally. The COVID-19 epidemic has caused havoc in this world, and through air-borne/physical touch infected millions of users. One of the most noticeable things was how almost everything went to be online, e.g., schools, businesses, and even our social life. This new situation created the urge for many people to have new “social life” to cope with the news, important updates, socializing with others, and, most importantly, work. Social Media Platforms (SMPs) like Facebook, Instagram, WhatsApp, and Twitter have been playing a major role since then. Studies had shown that the use of these social networks had increased and the way it was used is noticeably affected since the pandemic [1–4]. Not only has the use of social networks increased, but COVID-19 also became the most trending topic online in 2020 and cybercriminals have tricked users using news/information around COVID 19 to launch drive by download attacks and demonstrating another way COVID-19 has impacted our lives. These two factors encouraged cybercriminals to propagate malware through social networks using the topic of COVID-19 to attract people to their malicious content, especially Twitter, as the Twitter platform forces an auto-shortening to any included URL (that may lead to harmful web pages) within a 140-character message called “tweet”, so the URL will not be recognized if it was malicious. This paper gives an insight into hashtags related to COVID-19 that are used to redirect users to web pages containing malicious code. In the past few years, researchers focused their interest on the use of trending topics to spread malware over Social Media, each in its own way. The adaption of ML concepts and applying its algorithms is one of the proven effective approaches to detect, identify, and even block the propagation of malware. In this study, the main task of the ML models is to predict and classify imported tweets as harmful and safe. Therefore, the suitable models to use would be Classification Predictive Models, so we develop supervised ML classification models (Decision Tree, Random Forest, and Naïve Bayes) to find some similarity patterns in tweets that contain malicious URLs from a dataset collected from Twitter using the Twitter API and classify these URLs as harmful or safe using VirusTotal and again through the help of VirusTotal API. These developed models will later be tested on a new collected dataset to check the quality of their performance. Those approaches were high-quality performing models with high F1-score of 81%, 84%, and 72% and Accuracy of 92%, 94%, and 90% for Decision Tree, Random Forest, and Naïve Bayes, respectively, for the original collected dataset.

## Background and Literature

With the rising number of users for many Social Media Platforms (SMPs) (Facebook, Instagram, Twitter, WhatsApp, Telegram, etc.) in the past few years and the simple way to publish information, the possibility for cybercriminals to conduct their attacks, has increased too [5] and more tools and ways are being used nowadays to spread malware over these platforms. Malware (Malicious Software) is a piece of software that can take many forms of threats like viruses, worms, Trojan horses, etc., and it can be spread through emails, webpages, memory drives, and many other ways [6]. In this study, we are specifically looking at spreading malware through webpages presented by malicious URLs embedded within a text, that is, a malicious URL is a link that takes the user to a harmful domain, interacts with the user’s browser without the user noticing any suspicious activity, and then, the attacker can exploit vulnerabilities in the system and gain control over it [7, 8] which may lead into different kinds of cyber threats like ransomware, tacking over a financial account, or important information disclosure. According to the statistics from backlinko [9], until September 2021, more than 4.4 billion people are using social media globally, and this is an extensive base to be targeted by cybercriminals and spread malware [10]. For that reason, researchers have recently focused their interest on the propagation of malware through SMPs, and studies have shown that these platforms are being used to propagate harmful content and many users intendedly spread malware [11] and [12].

For cybercriminals to reach the larger possible number of people and lure them into engaging with malicious content, they tend to use trending topics or news that highly interest the public and include harmful content in them [7, 13]. Since late 2019, the COVID-19 pandemic had led the news and captured the interest of people all over the globe to become the #1 trending topic, and according to Twitter, the hashtag #COVID19 was the most used hashtag in 2020 besides other COVID-related hashtags [14]. On another aspect, a study was made by researchers at McAfee [15] showed a timeline for when the pandemic started, stating that there were subsets of common malware groups with high risk related to COVID-19 references. This timeline indicates how the rate of propagating these groups of malware increased since the pandemic. For that reason, we chose this trending topic to analyze and identify whether it has been used to propagate harmful content through SMPs.

Among the many SMPs, Twitter was chosen for this study, because (i) it is counted as one of the most popular platforms that influence the public view, and so many people are looking for important news through it [16], as governments and public figures use Twitter to announce exclusive news [17], (ii) according to the Washington Post, Twitter’s users’ records state that at the end of 2019, the daily online users are 152 million to rise to 166 million in 2020, recording the fastest growth rate in the platform users since 2016 when Twitter started reporting metrics and 24% higher than the year before [18]. Moreover, and the fact that Twitter is popularly used, and (iii) Twitter has a special platform for developers to gain unique access to Twitter’s content and use it for academic research and analysis purposes [19] through application programming interface API.

Some of the researchers made their studies trying to identify spammers on SMPs (users’ post-malicious content). In 2014, Soman and Murugappan made a two-direction study, showing that for some trending topics on Twitter, there have been spam tweets posted. The first direction was using the Fuzzy K-means (FKM) approach to cluster similar user profiles from collected trending topics tweets based on their extracted features. Second, they used extreme learning machine (ELM) for classifying the testing Twitter trending topics data as either spam or non-spam. Within the same research area, [12] showed in their research that suspicious users are spamming over SMPs. They identified several social user behavior-related characteristics from manually classified users as either to be spammers or non-spammers, and they used these characteristics as features for an ML process to classify a set of users to be either normal users (non-spammers) or spammers (tend to post-malicious content). Their approach correctly classified approximately more than 90% of non-spammers and 70% of spammers.

Similarly, [11] proposed a novel ML model; Supervised Spammer Detection with Social Interaction (SSDSI) which can detect spammers on Twitter based on the Content and Social Interaction, taking into consideration the social interaction frequency between users and their neighbors. In a study that was conducted by [20], three ML models were produced to detect spammers on Twitter based on user behavior features and some tweet characteristics. The researchers also proposed the best algorithm according to the performance results of each algorithm and showed how the performance can be enhanced if some features were eliminated. Stringhini [21] created honey profiles that would attract spammers on three SMPs and observed the attraction traffic, to later develop techniques that can recognize spam profiles. While, [22] developed an unsupervised ML approach to identify spammers on social networks.

Other researchers focused their work on the content that may be malicious rather than the user in SMPs. [23] developed a support-vector machine (SVM) algorithm to detect malicious content on Twitter, based on the analysis of language. Likewise, another study by [24] presented a system based on language features that spammers cannot easily manipulate. While, [6] were able to present a real-time malware detection approach on Twitter that gives an alert when a possible malware activity is active on the network. Furthermore, [13] presented an attack model that cybercriminals can carry out some attacks and infect other users even with a low connection degree.

### Machine Learning

Machine learning (ML) is a branch of artificial intelligence (AI), where the ML algorithms are continually developing and learning from the surrounding environment to gain knowledge based on given data features to mimic human intelligence and solve complicated problems [25]. A typical way to form a piece of knowledge and infer facts from data is by specifying some patterns in that data and predicting what would possibly happen or be. The automated version of the “knowledge-forming” approach is ML. An algorithm or a model takes input data with some additional features, identifies their unique patterns, and learns from them to make a decision or a prediction [26].

ML algorithms have been proven successful when applied to solve problems that rely on multi-relations features. These features can take many forms, like categorical, continuous, or binary, and they can be either unlabeled or labeled. When the features are unlabeled, the learning process is called unsupervised, and it is called supervised learning when the features are labeled [27]. Any ML input features are split into two types of variables for training. One is called independent variables which are all the characteristics the model will learn from and they are many. The second type is called the dependent variable and it is only one variable, which represents the true value that is to be predicted later.

Supervised Learning is creating patterns and general hypotheses based on the provided labeled features, and then making predictions for new future instances by learning from these patterns [28]. It is used when the model deals with a class-imbalanced dataset, that is, the training data have significantly different frequencies that will lead the model to rely on a sizeable classified part to be either positive or negative [29].

Supervised learning can solve either classification or regression problems depending on the features form [30], that is, when they are formed as categories; it is a classification supervised learning where the model job is to predict a class of an item, e.g., true or false, positive or negative, malicious or not malicious, or it is a regression when the features are continuous, and the model would predict an actual value of an item like prices, age, area, and so on. With the help of algorithms or what is called classifiers, Classification Supervised Learning takes sets of unseen data and categorizes them into classes based on the learning from the labeled features.

Supervised learning includes many algorithms like Naïve Bayes, decision trees, random forests, support-vector machine, neural networks, and so on [31]. In this study, the learning approach is mainly Classification Supervised Learning, and the algorithms that are going to be used are decision tree, random forest, and Naïve Bayes. The reason why classification is more suitable for this study is that we are dealing with imbalanced-class data that have an extensive range of features, and the potential output of the training would be either positive (tweet is malicious) or negative (tweet is not malicious).

#### Decision Tree Algorithm

**Fig. 1:**
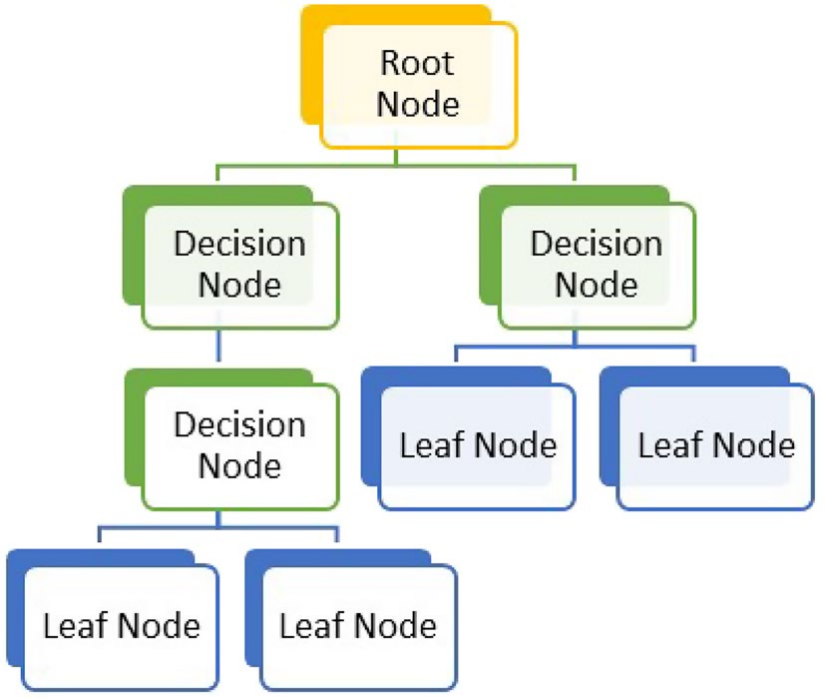
Decision tree structure

A decision tree (DT) illustrated in Fig. 1 is a supervised ML method that can be used for both regression and classification. It is based on a series of questions to classify the target variable by learning how to decide and infer the knowledge from the data features. A decision tree has the structure of a tree where there is a root node, decision nodes, and terminal node. The root node has the main role in the classification process, and represents the first question related to the problem where all the training instances are assigned and that will lead to decision nodes. Decision nodes are where the model checks for an answer (using if–then statements) to decide which is the best feature to take for the next step to construct a leaf node that represents one class until it reaches the terminal node where the final decision is made. When the DT model is fully trained, it becomes ready to take new unseen data and test it, and predicts the value of the target variable [32].

#### Random Forest Algorithm

**Fig. 2:**
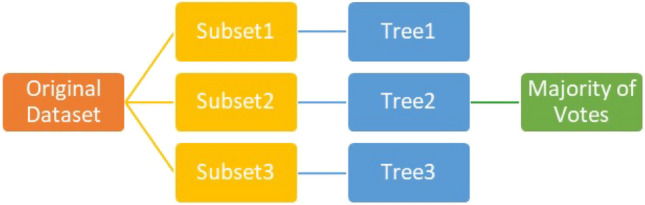
Random forest structure

A random forest (RF) is a supervised ML algorithm that can also be used in classification and regression problems. The need to use the Random Forest algorithm instead of DTs is when the DT model is overfitted (it performs very well with the training data and fails with the testing data). RF model (Fig. 2) works by making a number of decision trees (to be specified by the developer). Each tree takes a random subset from the original dataset to make a decision. These subsets may differ from the whole original dataset, where some may randomly drop rows or columns. After each tree gives its decision (vote), the RF model takes the average of the votes in case of a regression problem or the majority of votes when it is a classification problem. The ‘random’ assignment of data to the trees of the ‘forest’ makes the performance of each tree more intelligent and eventually avoids overfitting [33].

#### Naïve Bayes Algorithm

A Naïve Bayes (NB) algorithm is one of the most effective ML algorithms for classification problems, and it is robust, fast, highly scalable, and reliable.

It is a probabilistic model based on the conditional probability of an event, given that another event has already happened. In other words, it uses the probability of one event to predict the likelihood of another one. The following equation describes the theory behind the process in Naïve Bayes:

1 $$P\left( {H\left| X \right.} \right) = \frac{{P\left( {H\left| X \right.} \right) * P\left( H \right)}}{P\left( X \right)}$$

where *P* is the probability, *H* is the hypothetical event to be predicted, *X* is an already happening event, and *P*(*H*|*X*) is the probability of the event *H* to happen, given that the value of event *X* is true [32].

#### Evaluating Metrics

To ensure solving a given problem as good as possible, a model’s performance must be evaluated to decide whether it is good or not or to choose the best model among different models [29]. To evaluate the performance of any ML model, we calculate some values based on the comparison between the prediction results we got from each model and the original data values. These evaluating values will determine how accurate the model is, and which model among the others is the best.

*Confusion Matrix*: (Table 1) is an N×N matrix that summarizes the prediction results to later evaluate the performance of a classification model. One of the matrix’s axis represents the model prediction, and the other one represents the actual values. There are four possible states of the results based on the correlation between the actual label and the model’s prediction. A model can correctly classify an input value as True (true positive, TP), incorrectly classify an input value as True where it is actually False (false positive, FP), correctly classify an input value as False (true negative, TN), or incorrectly classify an input value as False where it is actually true (false negative, FN) [34].

**Table 1:**
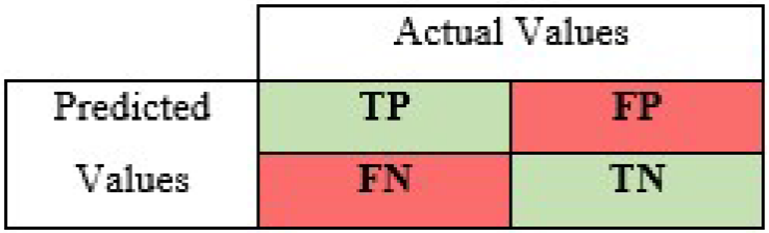
A confusion matrix

*Accuracy*: is the ratio of summation of the right predictions (true positive + true negative) out of the total predictions (true positive, true negative, false positive, and false negative). The higher the value of Accuracy, the better the performance of a model. It is a simple straightforward method to evaluate a model’s performance. However, when the dataset is a class-imbalanced dataset, the accuracy may not be enough to evaluate the model performance and extra measurements should be extracted to get a better evaluation of the model performance quality [35]. An example of that is if a dataset of 100 input values with the majority of them being negative (most frequent) and the minority is positive, and we can achieve high accuracy by simply assuming all new inputs to be negative, then the model performance accuracy will be very high indicating for excellent performance, while actually, this might be not the case [26].

*Precision*: is the ratio of the items that the model correctly classified as positive (TP) out of the whole correctly and incorrectly classified as positive (TP + FP) [35]. In other words, Precision captures the state where it is preferred to have TP values as much as possible [36].

2 $${\text{Precision}} = \frac{{{\text{TP}}}}{{{\text{TP}} + {\text{FP}}}}$$

*Recall*: is the sensitivity of the model and it is the ratio of the items that the model correctly classified as positive (TP) out of the correctly classified as positive and incorrectly classified as negative (TP + FN) [35]. As [36] stated in their book, Recall is the state where the preferred output is as many good or bad predictions as possible.

3 $${\text{Recall}} = \frac{{{\text{TP}}}}{{{\text{TP}} + {\text{FN}}}}$$

*F Measure or F1-Score*: is a value that measures the accuracy of a binary classification model performance [35]. It is the harmonic mean H of the precision and recall. The F measure (Eq. 1) is commonly used to evaluate the performance of ML models that deal with Natural Language Processing NLP, and it can be adjusted to give more importance to either precision or recall [37].

4 $$F_{1} = 2\frac{{{\text{precision}} \times {\text{recall}}}}{{{\text{precision}} + {\text{recall}}}}$$

### Natural Language Processing (NLP)

By the definition of the ML process, the ML models or algorithms take some features’ values as input to learn from and predict future unseen data. These features can only be of the numerical type, so that the model can take them as input [38, 39]. For this study, we work on in this study, the ML classification to be done is based on a collected dataset of tweets. These tweets are human language content that the ML models are not able to understand. To take out features from these tweets in a numerical form, natural language processing (NLP) is the approach to achieve that [40]. The main idea behind natural language processing is to transform the natural human language into a form that can be meaningful for computers to understand and process like numeric form.

As [39] explained in their book, NLP is a very challenging task to achieve, as it depends on human languages; that is, these languages introduce problems that differ from one language to another. Natural Language Processing works in many different ways presenting several applications like text summarization, part-of-speech (POS) tagging, Speech Recognition, etc. [39]. Each application gives different outputs to be processed and analyzed in solving problems. In the context of this study, as mainly we are focusing on the tweet text, we use NLP techniques to extract some valuable features that the ML models can understand. These features are POS-tagging and Sentiment Analysis, where the models will extract patterns based on them to infer and predict later which tweet might be malicious and which is not.

### Part-of-Speech Tags

Parts of speech are identified entities of a sentence that are helpful clues to better understanding the sentence’s meaning [41]. Part-Of-Speech Tagging or POS tags are the automated process of reading a text written in some language, analyzing it, and then assigning ‘tags’ to each word of the text to tell what ‘part of the speech’ is it [41, 42]. There are numerous POS tags [43] describing almost all kinds of words in a language. However, in this study, we use some of them that are representing the main parts of the speech as follows:

- CC: Coordinating conjunction (and, but, or).
- CD: Cardinal number (seventy sex, eight million, late 1970).
- DT: Determiner (a, the).
- IN: Preposition/subordinating conjunction (of, by, in, to).
- JJ: Adjective (smart, tall).
- MD: Modal (should, can).
- NN: Noun singular (car, dog).
- NNP: Proper noun (Cardiff, Ahmed, Friday).
- NNS: Noun plural (cats, mice).
- PRP: Personal pronoun (I, he, we).
- RB: Adverb (softly).
- RP: Particle (at, away, about).
- VB: Verb base form (eat, learn, teach).
- VBD: Verb past tense (ate, learnt, taught).
- VBG: Verb gerund/present participle (going, living).
- VBN: Verb past participle (gone, been, done).
- VBP: Verb not third person singular, present tense (write, spill, brush).
- VBZ: Verb third person singular, present tense (takes, looks, helps).

Through the results of the POS-tagging, we are going to use in this study, and the models will compose the similarities within the tags of the tweets as patterns and analyze them and gain the knowledge of whether these tags patterns differ in a malicious tweet from the ones in the normal tweets.

### Sentiment Analysis

Sentiment analysis, also called opinions mining, is one of the most commonly used techniques of natural language processing (NLP). It is a technical method to analyze text and study sentiment, opinion attitude, and emotions expressed within that text [44]. Sentiment Analysis methods can effectively measure feelings and thoughts for certain groups of people on selected topics. With sentiment analysis, we can quickly determine people’s impressions about any topic and decide whether they are happy or sad, positive or negative, and so on [4].

Billions of people all around the world share their thoughts, fears, stories, mental states, and many other moments every day [45]. And as mentioned earlier in this section, the main topic of the collected dataset of tweets is the COVID-19 pandemic. This pandemic as [4] explained in their study, had affected people all over the globe, and rose many conflicted feelings like stress, fear, and intensity. Therefore, we put a hypothesis that the emotions would make an important part of the textual input and may form a recognizable pattern for the ML models within the malicious tweets. Then, we check how accurate this assumption is and what is the effect of the sentiment of the tweets on the models’ performance results. We also assume that the 140 characters’ length limitation of a tweet forced by Twitter would make cybercriminals consider using the language carefully which would make a common pattern in the malicious tweets.

### Application Programming Interface

An API is a set of defined software functions that create an ‘interface’ to enable ‘applications’ to communicate and exchange functionalities and data with third-party developers and each other safely, by providing them with some credentials to make the connection happen. Many popular web applications nowadays are providing APIs [46].

## Methodology and Implementation

### Collecting Dataset

The dataset used in this study is collected from Twitter. Twitter provides a special platform called *Developers Platform* for researchers to do their studies based on Twitter content. This platform has the products (Twitter API, Twitter Ads API, Twitter for Websites, and Labs). These products are to be provided to developers, so they can connect to Twitter and stream a variety of different resources like (tweets, users, direct messages, lists, trends, media, and places). In our study, we use the Twitter API product by building a Twitter application on the platform to get the API credentials and stream ‘tweets’ in a JSON format.

In the first piece of code, we use tweepy python library. It gives the ability to conveniently access Twitter API using the four credentials (consumer key, consumer secret, access token, and access token secret) to live-stream tweets (and many other resources) and then write them into a JSON file. The stream should be authenticated by passing the API tokens through an object of the class OAuthHandler from tweepy, and it is filtered to stream tweets that are related to COVID-19 only. The filter is based on the given hashtags list we have created, which is consisted of 35 hashtags *isolating*, *isolation*, *selfisolating*, *self isolating*, *selfisolation*, *self* *isolation covid vaccine*, *covidvaccine*, *wearamask*, *wear* *a mask*, *stopthespread*, *stop the spread*, *covid*, *covid* *19*, *covid19*, *coronatextit*, *coronavirus*, *corona virus*, *stayhome*, *stay home*, *StayHomeSaveLives*, *covid-19*, *lockdown*, *quarantine*, *pandemic*, *covid19 pandemic*, *social distance*, *social distancing*, *SocialDistance*, *SocialDistancing*, *WFH*, *working from* *home*, *WorkingFromHome*,*work* *from home*, and *WorkFromHome*. Finally, the produced JSON file will go through the next stage as input for the second piece of code for pre-processing and producing the CSV file.

### Dataset Pre-processing

**Fig. 3:**
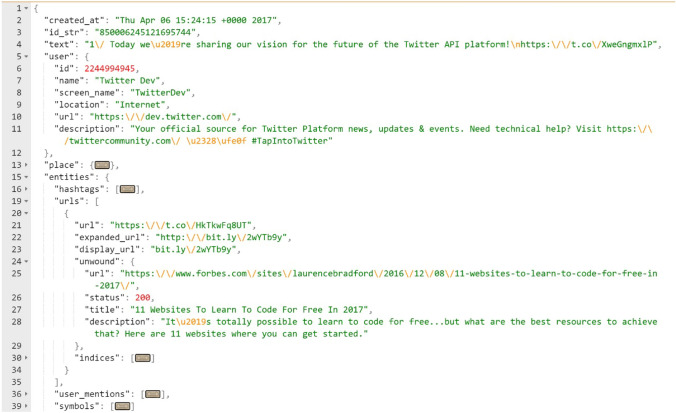
JSON structure of a tweet

The ML models basically rely on some features extracted from the original tweet text, user characteristics, and the URL within the tweet that will define the tweet as malicious or not. All Twitter APIs return encoded data using JSON. The JSON data format is very much similar to python dictionaries, and it has the structure of key-value pairs; these pairs are used to describe an object with attributes (keys) and values associated with them. Figure 3 illustrates the structure of a basic tweet in the JSON format. The main keys in the tweet shown below are’created at’,’id str’,’text’,’user’,’place’, and ‘entities’.

This format is not meaningful to an ML model as they take only numerical input, so it needs to be adjusted to another form, so that we produce the numerical features and the models can read them properly. The essential attributes that are to be used by our ML models are: (i) some tweets characteristics from the “text” key that holds the original tweet text in addition to the attributes to be created out of the text using NLP techniques, and (ii) attributes extracted from the key “user” that holds the needed characteristics of the user who published the tweet, and the “entities” key that contains the “hashtags” and “urls”, in addition to some other attributes that will be used in analysis and observation.

The final output result for the pre-processing is the initial CSV file consisting of 20 primary columns (Table 2), representing the basic features of every single tweet and 196,659 rows that contains all tweets and their corresponding features. It was later combined with another CSV file with the same columns layout to be 241,449 rows. This file will be the main input in the next step, that is, extracting the features from the tweet text using NLP techniques, and the values stating if each URL is safe or malicious, and these features will be used as independent variables for the ML models.

**Table 2:**
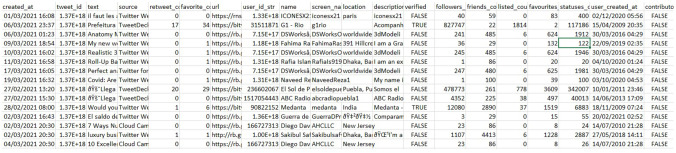
Initial CSV table

### Extracting Features and Finalizing the CSV File

The features that will be used as the independent variables for the ML models are content-based features and user-based characteristics, in addition to the value of the reports sent from VT stating whether the ‘expanded url’ is malicious or not, that is, this value will be the dependent or decision variable for the ML models.

#### Content-Based Features

There are two categories of features to be used as training features in the ML models:

• Features from the main structure of the JSON tweet:

- *Retweet count*: which is the number of how many times the tweet was retweeted.
- *Hashtags count*: is based on the ‘hashtags’ key that belongs to ‘entities’ key and shows how many hashtags were used in a tweet.

• Features that are extracted from the tweet ‘text’ by the python code:

- *Tweet characters count*: holds the value of the tweet length.
- *Words count*: states the number of words in a tweet.
- *Special characters count*: shows how many special characters are there in the tweet.
- *Numbers count*: shows how many numbers are there in the tweet.
- *URL length*: counting the ‘expanded url’ length.
- *Emotions count*: counting the occurrence of basic emotions (anger, anticipation, disgust, fear, joy, sadness, surprise, trust) in the tweet.
- *Tweet sentiment*: state whether the tweet is positive or negative.
- *Tweets Part-of-Speech (POS)*: counting how many verbs, nouns, adjectives, adverbs, etc. are in the tweet.

#### User-based characteristics

The user-based characteristics that were used as independent variables in the ML models are:

- *Friends count*: the number of friends of the user who published the tweet.
- *Verified*: a Boolean value (True or False) that states whether the user is verified by Twitter or not.

#### Piping URLs to VirusTotal

As explained previously, an ML model needs a dependent or a target variable beside the independent variables to accomplish the learning process and, hence, the prediction. In this study, the key feature which represents the dependent variable for the ML models is the value of whether the URL within the tweet is malicious or not, in which malicious = 1 and benign = 0.

**Fig. 4:**
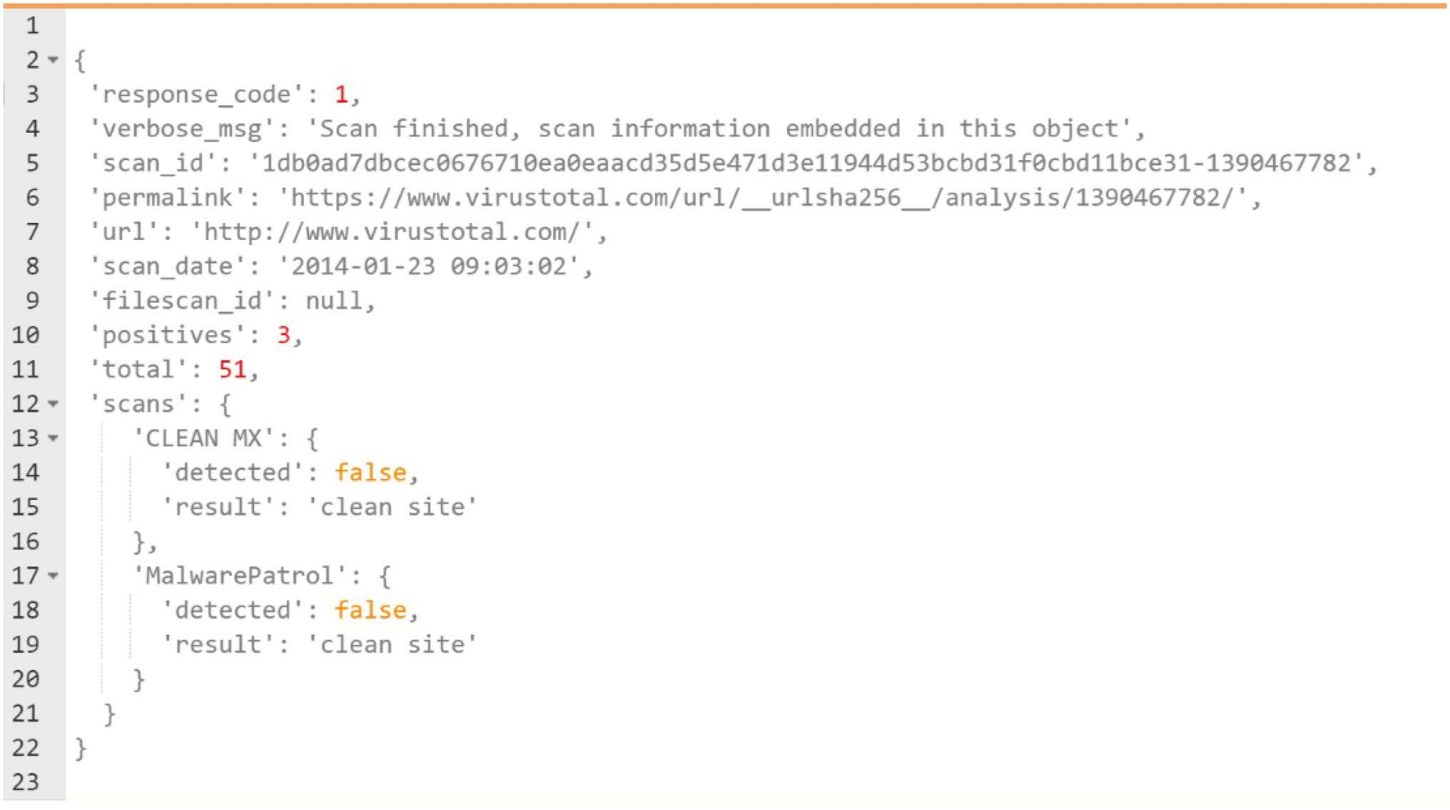
VT report structure in JSON format

To check the URLs, again, an API service from VirusTotal is used. As mentioned earlier, when passing the inputs through a code, the VT website sends back the response reports through the same code in a JSON format (Fig. 4). These reports can be read, processed or written into other files. The keys of the reports which indicate whether the URL is malicious or not, are: ‘response code’ which can either means that the requested URL is present in the VT databases if its corresponding value is 1 or not if its value is 0 and ‘positives’ that states how many times this requested URL was reported as malicious. As a result, if the values associated with these two keys are equal to or greater than 1, then the URL is malicious.

### Building the ML Models

In this study, three Supervised Classification ML Models are built, taking into consideration, the problem question and the number of features that were extracted to be independent variables. The dependent variable that the model will depend on to make the prediction is the value from the column called ‘is malicious’ of the dataset. Then, each model is evaluated by calculating the values of accuracy, F measure (F1 score), precision, and recall. Finally, dropping some of the features and see how the features elimination is going to affect the performance.

#### Developing the Classifiers

Earlier, we discussed how A Decision Tree (DT) works. We developed the decision tree model in the python code by importing the Decision Tree Classifier from sklearn library and all the corresponding evaluation metrics modules. As was explained before, ML algorithms can take data of numerical type only, so to avoid getting errors, we need to transform non-numerical data into numerical. Then, we assign *X* variable to all the independent variables column from the dataset which makes *X* a multi-dimension array, and *Y* to the one column of the dependent variable (‘is malicious’). Next, we split both *X* and *Y* into training data and testing data. For our approach, we take 60% of the data for training the model and the rest for testing the performance of the model. Then, we fit the training parts for both *X* and *Y* into the classifier. Now, to test the model, we create an object of the imported Decision Tree Classifier class and assign it to a variable through the predict() function that will take the testing part of the variable *X*. The final step is to evaluate the model performance by checking the evaluation metrics: accuracy, precision, recall, and F1-score. These metrics are already existing methods in the sklearn library, which we call and pass their parameters to be the original testing inputs and the prediction values associated with them.

The same approach is applied exactly the same for the Random Forest and Naïve Bayes models, except the used classifiers are RandomForestClassifier and GaussianNB, respectively. As was explained the random forest model basically a set of several decision trees, so when fitting the training data into the classifier, we need to pass the number of how many decision trees to be created in the random forest. There were three python codes for each ML model we used separately; ‘DT model.py’, ‘RF model.py’, and ‘NB model.py’. The python libraries for these codes are: pandas to read the dataset, and sklearn to import all necessary ML classes and modules.

## Results and Analysis

### ML Models’ Results

For testing the models, a random set of the final CSV file (instead of the whole dataset) was used as the input for the models, it consists of 120,046 rows of input, and as was described in the ML models pseudocode, 60% of the input dataset is used as the training data, while the rest 40% will be the testing data. The independent variables will be fit into the variable *X* and split into *x* train and *x* test, while the dependent (decision) variable will be fit into the variable *Y* and split into y train and *y* test.

#### Decision Tree Output Results

The DT model output results give the confusion matrix in (Table 3), where out of 48,018 inputs, the model correctly classified 40,319 tweets as malicious (TP) and 3995 as not malicious (TN), and it incorrectly classified 1945 tweets as malicious while they were benign (FP) and 1759 as benign when in fact they were not malicious.

**Table 3:**
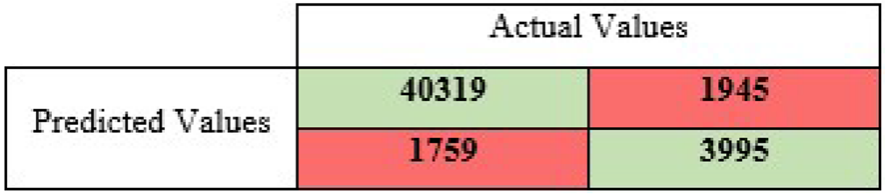
Decision tree confusion matrix

The numbers in Table 3 indicate the fact that the DT model was 92.2% accurate in classifying the tweets, which is a sign of a very good performance. However, as mentioned in section two, to properly evaluate a performance of a model with a class-imbalanced dataset, extra measurements need to be done. Therefore, we check precision, recall, and F1-score. The DT model gave Precision = 81.53%, Recall = 82.41%, and F1-score = 81.96%. By the definition of these terms, these results are indicating to a very good performance of the ML model and the model can be relayed for predicting new unseen data. When we randomly dropped out some of the features of the independent variable, there was a slight increase in the model performance of approximately 0.3%. On the contrary, when we intendedly to drop some features based on the hypothesis, we adopted earlier of how the tweet length and emotions may matter the evaluation metrics dropped where the Accuracy decreased to 88.6% from 92.2%, the precision dropped to 72.9% from 81.5%, Recall decreased by 13 points to become 69.4%, and finally, the F measure came down to 70.9% from 81.96%. Moreover, a similar performance drop occurred when we eliminated only the URL length attribute itself, to have the values Accuracy = 89.1%, Precision = 74.2%, Recall = 75.02%, and F1-score = 74.6%. These results indicate that the eliminated features that caused decreasing the model performance are important features composing the key patterns of a malicious tweet.

#### Random Forest Output Results

**Fig. 5:**
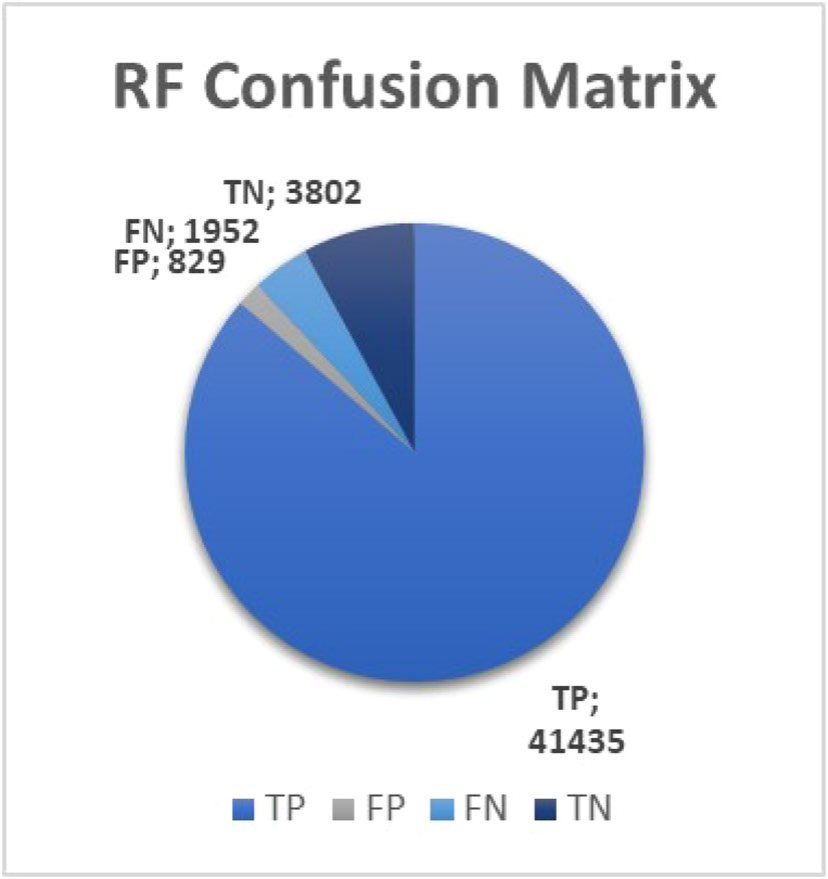
Random forest confusion matrix

As shown from the confusion matrix in (Fig. 5), the numbers indicate to a high-quality performance for the Random Forest model. As it has been able to correctly classify 45,237 tweets out of 48,018, meaning that the accuracy of the model is 94%. However, as the model is relying on features that have different ranges of frequencies, e.g., ‘friends count’, ‘tweet length’, ‘url length’, etc., we look at the other evaluation metrics. The model had Precision = 88.79%, Recall = 82.05%, and F1-score = 84.98%, as the TP = 41,435, FP = 829, TN = 3802, and FN = 1952. According to the numbers, again, we have a high-quality performing model that can be relied on to predict new entries.

Following the same approach, we used in the DT model testing the elimination of the features that we inferred to be important, decreased the quality of the Random Forest model too. The evaluation metrics dropped by 5–16 points to become as follows: Accuracy = 89.8%, Precision = 77.4%, Recall = 66.5%, and F1-score = 70.1%. Furthermore, as explained in the methodology of building the ML models, a random forest consists of multiple decision trees and we need to specify how many trees the random forest has. In our approach, we specified five trees. Generally, when we increased the number of decision trees in our RF model, the performance increased.

#### Naïve Bayes Output Results

When running the ‘NB model.py’, the model uses the input dataset and randomly divides it into multiple subsets to make the final decision. The Naïve Bayes model was able to correctly classify 40,810 tweets as malicious and 2407 as benign. On the other hand, it wrongly classified 1454 tweets as malicious when, in fact, they were benign and 3347 were malicious but classified as not. These numbers mean that the model is 90% accurate for our study dataset and it has Precision = 77.38%, Recall = 69.19%, and F1-score = 72.25%. A model with these metrics can surely evaluate as a good performing model. Similarly to Decision Tree and Random Forest models, the performance quality of the Naïve Bayes model decrements when we delete the same features we deleted in the previous models. Therefore, the new metrics values became Accuracy = 88.7%, Precision = 76.2%, Recall = 57.04%, and F1-score = 59.2%.

#### Evaluating the Best Model

**Fig. 6:**
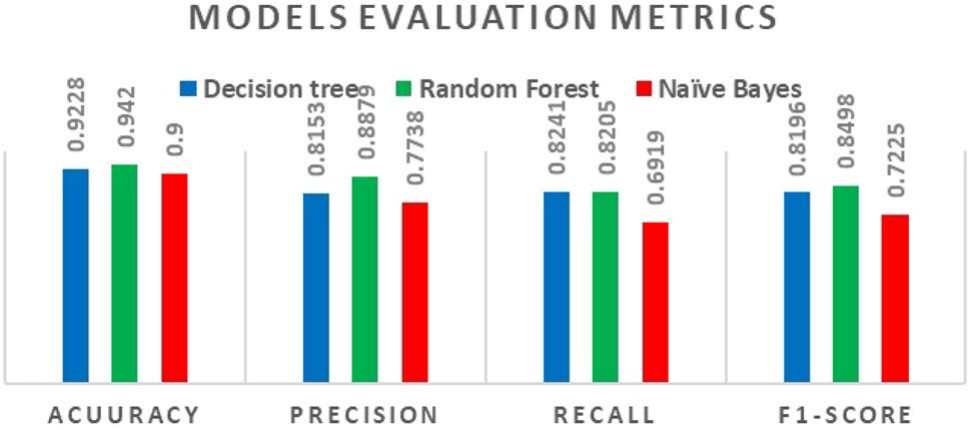
ML models’ evaluation metrics

**Fig. 7:**
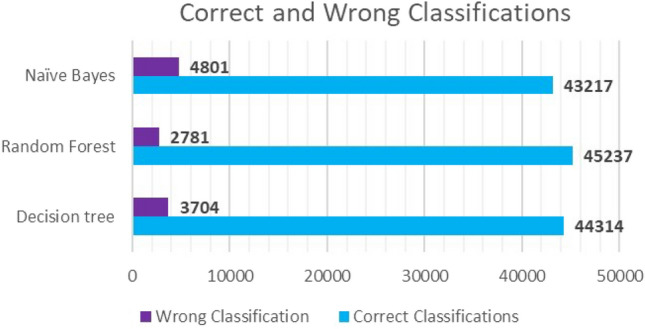
Correct and incorrect classifications for each model

The evaluation metrics (accuracy, precision, recall, and F1-score) are illustrated in the chart below in Fig. 6. Overall, the three models performed very well and there are slight differences between the three selected models in performance quality. As mentioned earlier, the higher the metrics values are, the better the model is. According to the stated results in the previous sections, the best classifying algorithm overall is the Random Forest algorithm which has an accuracy of 94%, while DT and NB algorithms have an accuracy of 92% and 90%, respectively. The same inference is true even when manipulating the input features of the three models. Looking at the ratio of the correctly classified as malicious tweets over the whole classified as malicious (Precision), again random forest has the highest value of 88% which is 11% and 7% higher than Naïve Bayes and Decision Tree algorithms, respectively; see Fig. 7.

### Is COVID-19 Being Used to Spread Malware?

Looking at the results from VirusTotal reports corresponding to our collected dataset of tweets that are related to COVID-19 only, we can state the following facts:

• Out of 241,449 COVID-19-related collected tweets, 31,689 tweets were reported positive for potential malware. Which is 13.12%.
• Based on the results from the trained supervised ML models, these tweets that contained malicious URLs have certain patterns. These patterns helped the models to quite accurately classify testing data.

Consequently, the topic of COVID-19 has been used by cybercriminals to attract people’s attention and spread malicious content to conduct cyberattacks. Taking the advantage of the sensitivity and importance of this topic to people and the fact that all URLs would not look suspicious as they are automatically shortened by Twitter even if the URL is already short.

### Dataset Observations and Statistics

Earlier, we discussed the actual numbers of our codes and how the ML models performed when testing them. Now, we look at some observations on the main dataset we have used in this study.

**Fig. 8:**
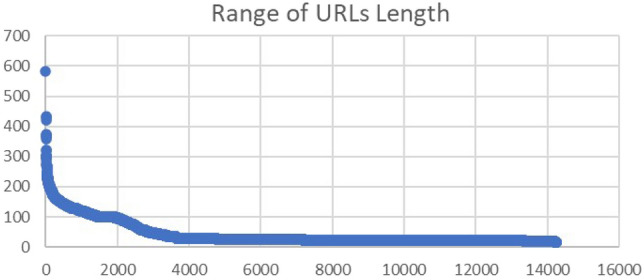
Range of malicious URLs’ lengths

1. There are some users that repeatedly published different malicious content at different times or even the same tweet but on different occasions.
2. Some malicious tweets were posted at the same time (or with a slight difference) by different users and from different locations. Which may indicate an automated method for publishing these tweets.
3. Some users tend to include the same malicious URL many times in different tweets taking the advantage of the automated shortening of the URL by Twitter, as the same URL is reshaped differently every time and it would be thought of as a different URL for a different topic.
4. In general, malicious tweets tend to have longer sequence of characters than the safe ones.
5. The URLs in the tweets classified as not malicious are generally longer than the harmful URLs (that are mostly short). In our dataset, the longest URL was composed of 1329 characters and it is a benign URL. While the longest malicious URL was of length 582. Figure 8 illustrates the range of malicious URLs’ lengths in the testing and training data.

### New Datasets and Statistics

Finally, a new dataset of tweets was used as the testing data and the whole previous dataset as training data. Testing the new dataset showed the following:

1. 31.1% of the tweets are malicious and 86.9% of them are not.
2. 52% of the tweets had positive sentiment.
3. 12% of the users that posted malicious tweets had verified accounts by the Twitter platform.
4. The top three counted emotions in the malicious tweets are: joy at 42.6%, fear at 23.8%, and lastly sadness at 12.8%.

**Fig. 9:**
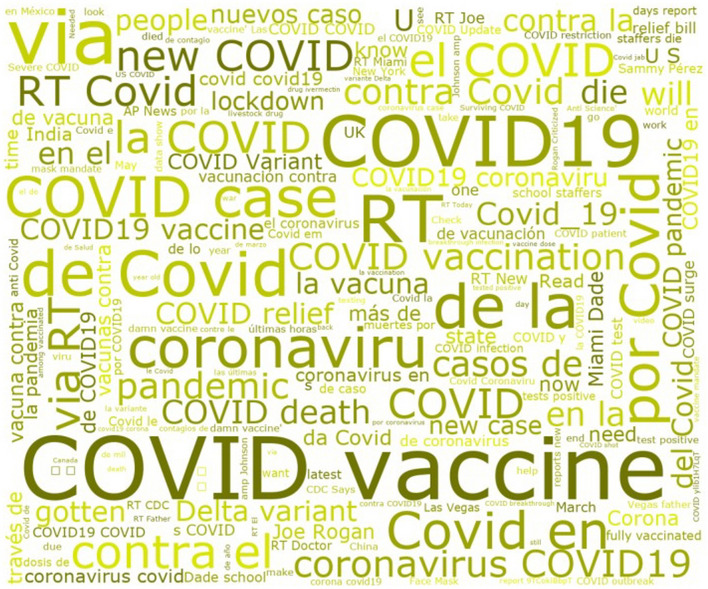
Word cloud image of the tweets text

A Word Cloud (Fig. 9) was generated based on the tweets’ text and showed that the most noticeable repeated words are (COVID, COVID-19, COVID, coronavirus, vaccine, death, vaccination, pandemic, etc.).

**Fig. 10:**
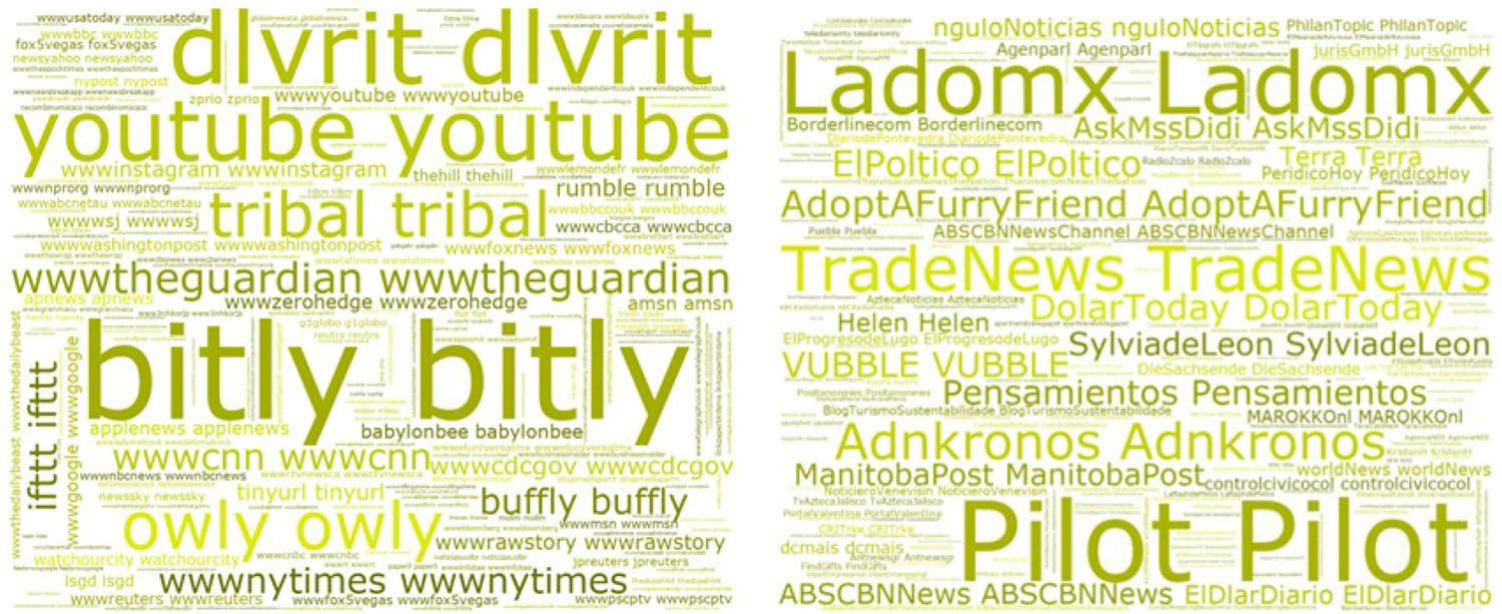
Word cloud of most frequent malicious domains and accounts

The results showed as discussed in the dataset observations that there were more frequent malicious domains and accounts that post-malicious tweets within the scope of our collected dataset (Fig. 10, with the Repeated Domains on the left and Repeated Users on the right).

## Conclusion

In this study, three Classification Supervised ML Models were developed, that is, these models are able to classify a set of tweets collected from Twitter (related to the trending topic of COVID-19 and contains URLs), besides with their corresponding features, as either malicious or not malicious tweets. The tweets were collected using Twitter API depending on a COVID-19-related hashtags list. They were live-streamed and saved as JSON files. Then, they were pre-processed and cleaned to be prepared for the later steps of the study. The result of the pre-processing was a CSV file containing the tweets and the basic information corresponding to them like creation date, location, text, user profile information, entities (URLs and hashtags), retweet info, and so on.

Then, we used Natural Language Processing NLP techniques to extract new features for the ML algorithms like tweet sentiment, emotions sentiment, and Part-of-Speech (POS) tags. We used NLP considering that the main structure of the study material is the tweet, which is a natural human language. The URLs within the tweets were tested in VirusTotal website using API.

The used ML algorithms were decision tree (DT), random forest (RF), and Naïve Bayes (NB). The models were evaluated for their performance using the validation metrics; Accuracy, Precision, Recall, and the F measure. In general, the results of each model indicate that these models are reliable and their performance was very good as the evaluation metrics were: Accuracy = 92.2%, Precision = 81.53%, Recall = 82.41% and F1-score = 81.96%, Accuracy = 94%, Precision = 88.79%, Recall = 82.05%, and F1-score = 84.98%, Accuracy = 90%, Precision = 77.38%, Recall = 69.19%, and F1-score = 72.25% for decision tree (DT), random forest (RF), and Naïve Bayes (NB), respectively. Even when the performance quality dropped when we eliminated some features, the results remained relatively good. According to these results, the Random Forest algorithm was the best-performing model among the others, because it was the most responsive model to features manipulating. We were able to identify important features from the ML variables, e.g., the emotions and tweets’ sentiment, the length of tweets, and the length of the actual URL within the tweet where evaluation metrics of the developed models decreased between the range of 5–16 points. We also noticed that for our study, the Random Forest model performance increases when we increase the number of decision trees in the forest.

We have also programmed a simple GUI tool with python that can take new unseen datasets as a file path string to read it and test it with the ML models we have previously developed. This tool visualizes some results taken from the training outcome as interactive charts and summarizes the most used words in the tweets, the most frequent malicious domain and the most frequent user account that posts malicious content within the dataset as images of word clouds. Although the performance evaluation of the models on the unseen data was less than the evaluations for the same original dataset, it still indicates to good performance and can be trusted.

Moreover, according to the observations made out from the CSV file, in general, the tweets’ sentiment was mostly more positive than it is negative, and the top three emotions noticed in the malicious tweets were joy, fear, and sadness. There were 12% verified users accounts out of the whole users who tweeted malicious content. And many of these users appeared more often than other users with many different malicious tweets. Some spammer on Twitter posted the same harmful link in many different posts, taking the advantage that every time Twitter shortens the link, it will take a different shape and cannot be recognized even by the trained eye.

An indication of the possibility of posting harmful tweets automatically by fake accounts was noticed when the same harmful tweet was posted at exactly the same time by different accounts and from different locations. It was also noticed that the malicious tweets have the longest possible sequence of characters while the malicious links tend to be shorter than the normal ones. Eventually, we can conclude that, according to the methodology we created and the way we implemented it and the results we got from both the actual ones from VirusTotal and the predictions from the ML we have developed and the collected dataset in specific, the topic of COVID19 was used to spread malware over Twitter.

To sum up, we were able to develop ML models with a high-quality performance depending on many content-based and user profile-based characteristics and build a visualizing tool to test a whole new dataset. We were able to indicate actual harmful content and put suggestions to resolve them, and these suggestions have been communicated to Twitter to help have a safer community on the platform.
